# Semantic segmentation of light-toned veins in multimodal ChemCam data

**DOI:** 10.1038/s41598-026-47207-0

**Published:** 2026-04-09

**Authors:** Ana Lomashvili, Kristin Rammelkamp, Protim Bhattacharjee, Olivier Gasnault, Elise Clavé, Christoph H. Egerland, Susanne Schröder, Travis S. J. Gabriel, Ari Essunfeld, Stéphane Le Mouélic, Begüm Demir

**Affiliations:** 1 German Aerospace Center (DLR), Institute of Space Research, Berlin, Germany; 2 Institut de Recherche en Astrophysique et Planétologie IRAP, Université de Toulouse, CNRS, CNES, Toulouse, France; 3 BIFOLD and TU Berlin, Berlin, Germany; 4 U.S. Geological Survey, Astrogeology Science Center, Flagstaff, AZ USA; 5 Los Alamos National Laboratory, Los Alamos, NM 87545 USA; 6 Laboratoire de Planétologie et Géosciences, CNRS UMR 6112, Nantes Université, Univ Angers, Le Mans Université, 44000 Nantes, France

**Keywords:** Chemistry, Mathematics and computing, Optics and photonics

## Abstract

Since the Mars Science Laboratory landed in 2012, the ChemCam instrument aboard the rover has collected in-situ laser-induced breakdown spectroscopy (LIBS) data and context images along more than 35 km of the Gale Crater traverse, providing valuable observations including diagenetic features such as light-toned veins. These veins are of particular scientific interest because they are interpreted as indicators of past fluid circulation on Mars and provide insights into the evolution of habitability on Mars. Their identification, however, currently relies on manual visual inspection of Remote Micro Imager (RMI) images, a process that is time-consuming and sensitive to differences in human interpretation. To address this issue, in this paper we introduce a novel pixel-level labeled, multimodal dataset of ChemCam observations specifically tailored for vein detection, along with customized U-Net models to integrate both textural (RMI) and chemical (LIBS) modalities. To further ensure trustworthy scientific use, we incorporate the Learn-Then-Test (LTT) framework to provide statistical control of the false discovery rate without requiring model retraining. The experimental results demonstrate that the proposed customized U-Net models trained on the developed dataset, combined with risk-controlled prediction, increases the efficiency of pixel-level vein identification through automation and produces statistically reliable predictions for multimodal ChemCam data.

## Introduction

Since 2012, NASA’s *Curiosity* rover has been exploring Gale crater on Mars with its central mountain Aeolis Mons (informally known as Mt. Sharp) whose layered sedimentary strata contain evidence of past environmental changes^1^. This landing site was chosen to search for evidence of past habitability, which could be confirmed early on in the mission^1,2^. Since then, *Curiosity* has continued to characterize the evidence of this ancient habitability in the rocks mainly with respect to aqueous conditions^3^. The rover is equipped with multiple scientific instruments. One of the instruments that studies rocks and soil on Mars is ChemCam (Chemistry and Camera)^4,5^. The instrument serves two functions: a Laser-Induced Breakdown Spectrometer (LIBS) and a Remote Micro Imager (RMI)^6^. LIBS employs a pulsed laser aimed at the target to create a light-emitting plasma, whose emission is collected and directed to spectrometers. The spectra contain information about the target’s elemental composition within a spot size ranging from 350 to 550 micrometers^5^. Prior to and after LIBS measurements, the RMI captures context images of the targets at sub-millimeter resolution^6^. The RMI images depict soil and different types of rocks, some with various diagenetic features^7,8^. Among those are features ranging from a few millimeters to several centimeters wide, that are rock fractures filled with the material identified by ChemCam to be calcium sulfate most of the time, which we refer to as “light-toned veins” herein^9–11^.

Light-toned veins on Mars are a subject of interest due to their link to multiple diagenetic processes, which involve chemical or physical alterations caused by fluids after the host rock formed, including mineral precipitation within fractures. From orbit as well as in-situ, multiple instruments detected calcium sulfates in different states of hydration^9,10,12–15^. Considering that these sulfates form primarily in the presence of water and tend to be evaporitic minerals, light-toned veins are believed to be the result of remobilization, evaporation, and precipitation during aqueous activity on Mars^16^. This made light-toned veins a promising diagenetic feature for investigating signs of past habitability, especially since they are present almost everywhere on this site. Furthermore, studies showing the correlation between veins and high calcium abundances motivate exploration of RMIs at the pixel level to match the appropriate spectral data^10,17,18^.

Since *Curiosity*’s landing in 2012, the growing size of collected and returned RMI data has increased the need for methods that can extract statistical trends and synthesize observations along the rover’s traverse. Given the scientific motivation to identify fine-scale diagenetic features in ChemCam targets, a few automated methods have been explored to support more systematic target selection and analysis. One of the earliest works is the autonomous targeting software AEGIS^19^. Operating on the *Curiosity* rover since 2015, the software suite not only selects targets for ChemCam, but is able to locate fine-scale features such as veins on rocks and adjust the pointing of the LIBS instrument accordingly^20^. The software suite operates with two algorithms: Rockster and TextureCam. Rockster is responsible for identifying targets in Navcam (Navigation Camera) images, and its operation is based on image processing approaches, while TextureCam uses a random forest classifier that identifies regions of interest in ChemCam targets, highlighting features such as veins or concretions, for precise laser pointing during LIBS measurements^20,21^.

Other works have applied machine learning and ML-adjacent methods to characterize and classify ChemCam targets by their associated RMI images. In one such study^22^, 17 visual attributes (bumpy, light-toned, coarse-grained, “has veins”, etc.) were identified from human visual inspections of ChemCam targets measured during the first 600 sols (martian days) of *Curiosity*’s traverse. Later, the images were grouped using various graph algorithms based on manually labeling each image with either “yes” or “no” to indicate the presence of the 17 attributes. Another work^23^ identified 9 image classes (fractures, veins, smooth, etc.) using unsupervised machine learning and applied transfer learning to automatically classify ChemCam targets into those groups using only images. A more thorough analysis of ChemCam targets would involve labeling each diagenetic feature at the pixel level, with the potential to compare textual information with chemical composition.

**Fig. 1:**
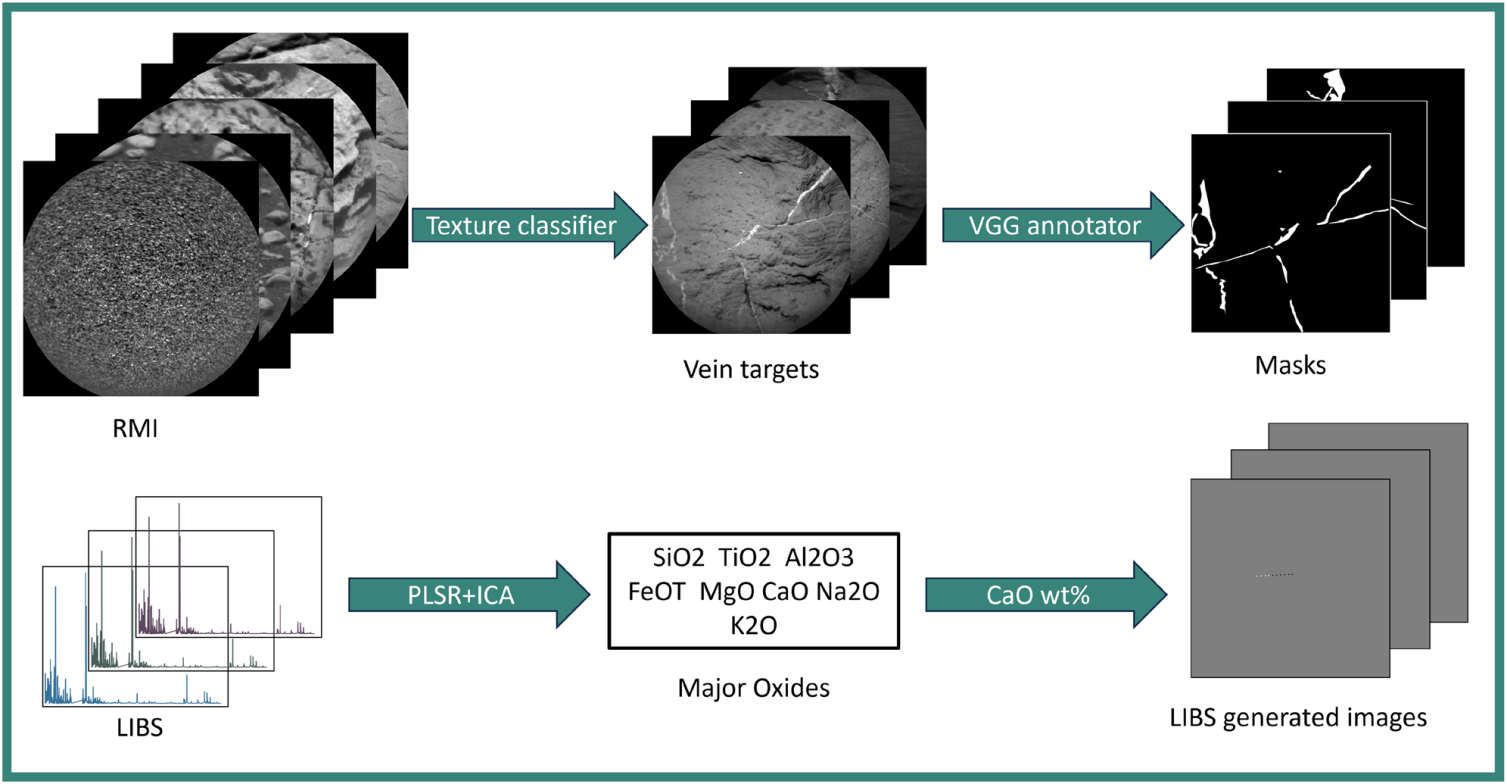
Workflow for preprocessing ChemCam multi-modal data. RMI images are processed with a deep learning texture classifier^23^ to predict vein targets, and corresponding masks are subsequently generated using the VGG annotator tool. Major oxide abundances are derived from LIBS spectra using a combination of partial least squares regression and independent component analysis (PLSR–ICA)^24^. LIBS generated images are then produced from the weight percent of CaO.

In this study, we develop a novel pixel-level labeled, multimodal dataset of ChemCam observations specifically tailored for light-toned vein detection in RMI images. An overview of the preprocessing steps applied to the ChemCam’s multimodal data is summarized in Fig. 1 and is described in detail in the following sections. Further, we present customized U-Net–based deep learning models for semantic segmentation of light-toned veins that integrates LIBS and RMI data^25^. By providing pixel level locations of light-toned veins, which previous studies have shown to be predominantly composed of calcium sulfate, in ChemCam RMI images, the model enables traverse-long statistical studies informing the role of water in the ancient climate of Gale crater, Mars^3,10,11^. In addition, we explore multiple strategies for integrating elemental information derived from LIBS into the RMI context images. For comparison, we also evaluate the transformer-based DINOv2^26^ architecture and the random forest-based TextureCam classifier^27^. To enhance the trustworthiness of the model’s predictions, we incorporate an application-specific risk-control mechanism that operates without requiring model retraining. Together, these developments result in an effective pixel-level detection method for light-toned veins in RMI data that can be flexibly adapted to user-defined requirements. This allows checking the chemical composition of the light-toned veins and supporting the identification of their type (e.g., evaporitic Ca-sulfates vs. igneous/anorthitic veins) by comparing the chemical data provided by the LIBS analysis with the locations of the veins predicted by our pipeline. At the rover’s traverse level, this is an efficient way to process all targets, characterize vein geometry, and analyze compositional variations of this diagenetic feature in all the mission data. Overall, we combine multi-modal data acquired by ChemCam in a systematic, statistical approach, enabling the characterization of Ca-sulfate veins in an efficient and reliable way, supported by formal statistical guaranties.

## Dataset and methods

### Dataset

The ChemCam instrument aboard the *Curiosity* rover employs LIBS to analyze the elemental composition of rocks and soil in Gale Crater, Mars^4,5^. The instrument shoots a laser at targets up to 7 m away, ablating material, and creating plasma. The emission of the plasma is then collected and transferred to spectrometers covering 240–850 nm^4^. Analysis of the acquired spectra enables determination of the elemental composition within a small spot on the target, approximately 350–550 $\upmu$m in diameter^5^. Measurements involve 30 laser shots per point. To account for potential dust on the target, the first five measurements are excluded, and the remaining measurements are averaged to calculate the elemental abundance for each point. Per target, ChemCam takes multiple measurements in small rasters with typically five to ten locations to monitor the variation in chemical composition. The quantification of major oxides (SiO$_{2}$, TiO$_{2}$, Al$_{2}$O$_{3}$, FeO$^{\text {T}}$, MgO, CaO, Na$_{2}$O, and K$_{2}$O) of each LIBS point is done with a multivariate regression model for which a balanced combination of partial least squares regression and independent component analysis (PLSR–ICA) is used^24^. As mentioned above, ChemCam also takes high-resolution context images with RMI before and after LIBS measurements^6^.

The ChemCam instrument has taken LIBS and RMI measurements of more than 4000 targets. The size of the images captured by the RMI is $1024 \times 1024$ pixels with a field of view of 20 $\upmu$rad^6^. The scale is subject to variation due to changes in the distance between the camera and the target. The images are taken in grayscale.

As an initial step, we feed RMI images, provided in the *NASA PDS Geosciences node*^28^, to the texture classifier, which is a preceding work of this paper^23^. The model classifies RMI images into 9 classes (veins, layered, smooth, low nodular, high nodular, fractured, soil, pebbles, drill) with $96\%$ of accuracy for the class of veins. We retrieve images where the model predicts the presence of light-toned veins, summing up to 480 targets, and subsequently perform visual inspection to remove false detections. Only images captured before LIBS measurements are selected to mitigate the potential impact of LIBS pits on the visibility of veins within the targets. In total, 55 images were manually annotated for model training, validation, and test. The selected set of images clearly displays light-toned veins.

**Fig. 2:**
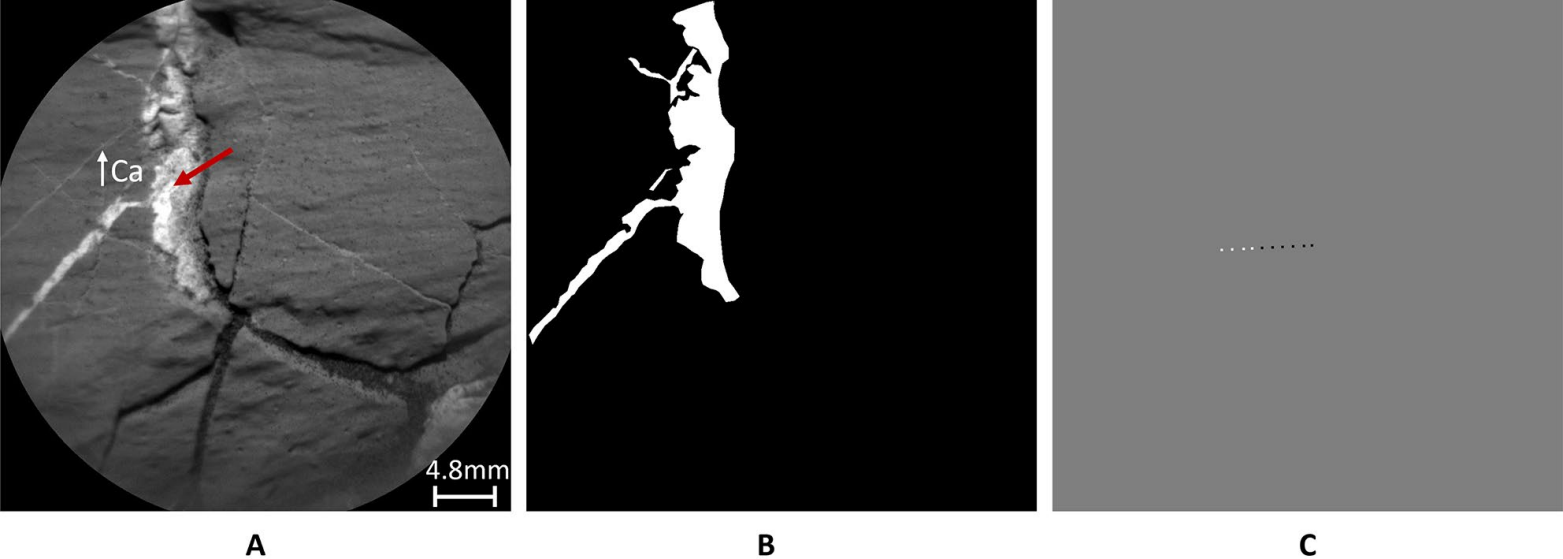
(**A**) is an example RMI image with Ca-rich light-toned veins from sol 2744. The red arrow points to the light-toned vein and indicates high Ca content. (**B**) is a manually generated mask (ground truth annotations for light-toned veins) for the RMI image in the left. (**C**) represents LIBS generated image for the same RMI image. In the gray area no LIBS measurements were done. The white and black pixels indicate high (>10 wt%) and low (<10 wt%) Ca-Oxide content accordingly.

To create masks, an open-source image annotation tool, VGG Image Annotator (VIA)^29^, is used. The vein regions are manually annotated and later colored with white pixels. The rest of the area is flooded with black pixels, resulting in a binary mask. A simplified schematic of the preprocessing workflow for RMI images is shown in the top panel of Fig. 1. The training, validation, and test sets have respectively: 40, 10, 5 images. In order to augment the training dataset, a two-step approach is employed. In the first step, the original image shape of $1024\times 1024$ pixels is divided into 16 equal tiles each, resulting in 880 images. Later, the training set tiles are fed into the geometric augmentation module, in which they are randomly flipped, rotated, and zoomed in^30^. In this way, the training set is prepared to be fed into a semantic segmentation model. Figure 2 shows the two inputs for training. Image **A** represents an RMI image fed to the model for learning features, and image **B** is a manually generated mask (ground truth) used in the loss function to train the model.

As mentioned previously, light-toned veins in ChemCam targets have been characterized as calcium sulfate with LIBS^10^. This allows us to use the high Ca content identified in LIBS spectra as further, though not unique, evidence for the presence of light-toned veins. Such additional information has the potential to refine vein segmentation models that employ only RMI images. First, we analyzed previous findings to estimate the appropriate Ca abundance, which indicates the presence of light-toned veins. In most studies, the chemical composition of targets is derived from the LIBS spectra for the major oxides, which are available in the NASA PDS Geosciences node^28^. The range of CaO in ChemCam targets varies between $[0-51.7]$ wt%. Studies presenting average bedrock compositions usually exclude LIBS measurements with high CaO abundances to avoid contributions from diagenetic features such as calcium sulfate-filled fractures, e.g,^31,32^. By doing so, chemical composition of the bulk rocks in different regions of Gale crater showed an average CaO abundance of less than 7 wt%^31,33–35^. Furthermore, an unsupervised clustering study of ChemCam LIBS spectra grouped measurements with similar composition^17^ and RMI images of a few samples taken from the only group showing elevated CaO abundance (median 21.5 with standard deviation 8.6 wt% CaO) in this clustering study confirm the presence of light-toned veins. Additionally, the provided average CaO abundance for all the other clusters showed less than 6.68 wt%. Following the review of previous studies and the distribution of CaO abundance along the traverse, $10\,wt\%$ of CaO or above is considered as an indication of potential presence of light-toned veins for the purpose of this study. Other compositional indicators from the literature are not incorporated in this study. The threshold of CaO $wt\,\%$ is not only used for comparison of the results but also to generate images showing occurrence of light-toned veins in LIBS locations. In Fig. 2, image **C** on the right, shows an example of the LIBS generated image. The image displays gray pixels in the area where no LIBS measurements were done. White pixels show CaO content greater than 10 wt% in the location of LIBS measurements and the black pixels indicate lower CaO abundances. The pixel-wise sizes of the LIBS locations were estimated by visual inspection of the LIBS pits visible in the RMI images taken after the LIBS measurement. Although the scale of the images is subject to change, the area of LIBS locations were fixed and represented $5 \times 5$ pixels, consistent with the pixel field of view (20 $\upmu$rad). For a target at 2.5 m, this corresponds to 250 $\upmu$m, comparable to the laser spot size^5^. The workflow for generating LIBS images is summarized in the bottom half of Fig. 1.

**Fig. 3:**
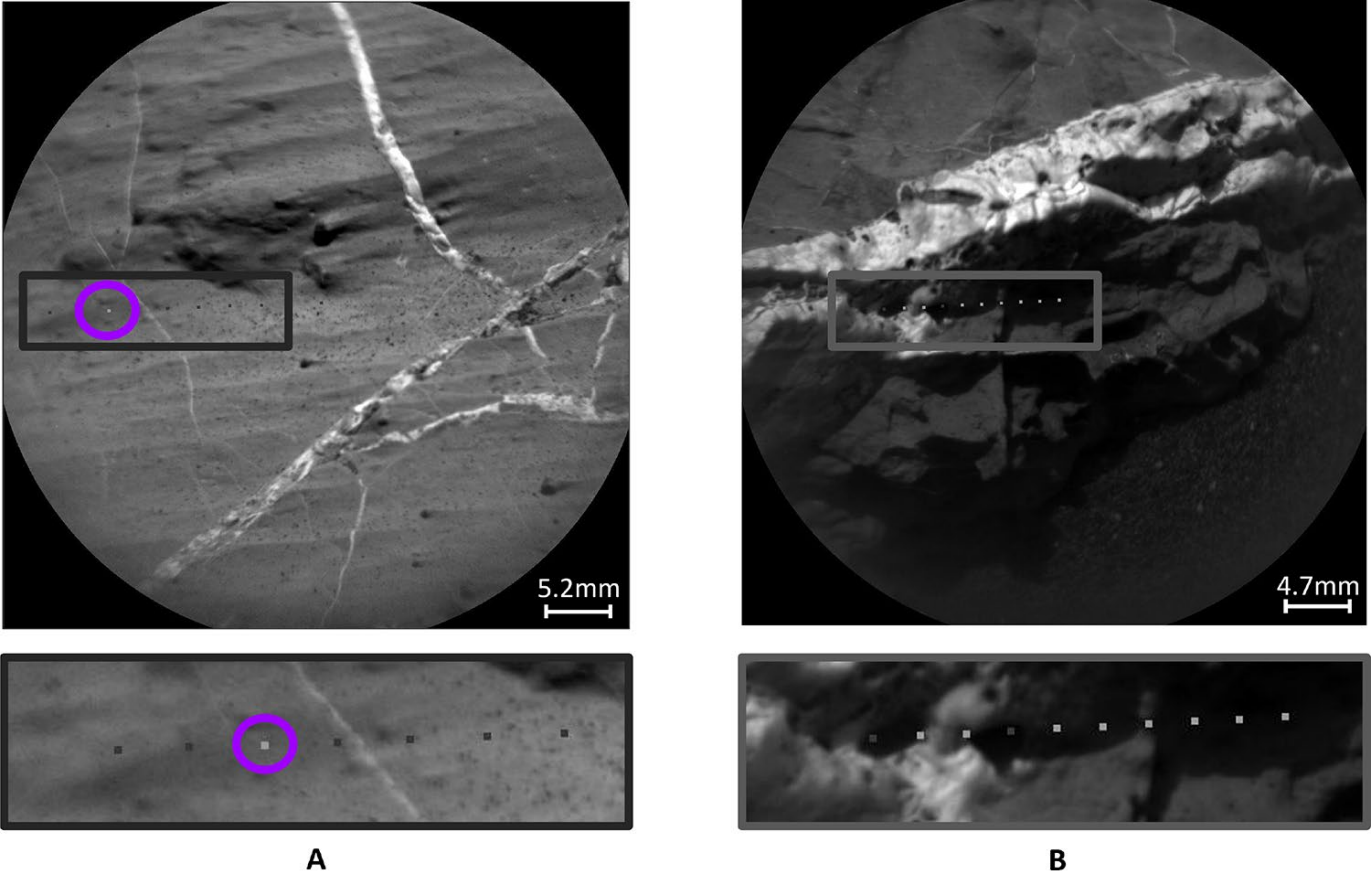
RMI images with zoomed-in areas below showing calcium abundances based on LIBS spectra, where darker squares indicate low calcium levels and lighter squares indicate high calcium levels. (**A**) Target Corpach sol 2744 shows high calcium levels at the point circled in purple, representing a potential false positive detection of light-toned veins based only on LIBS measurements. (**B**) Target Waku Kungo sol 1441, example of challenging lighting conditions, where light-toned veins can be hidden by shadow; LIBS points in these shaded regions exhibit elevated calcium abundance.

The accuracy of the models is estimated using the manually labeled data. Due to the challenges of accurately labeling images on a pixel level, the masks that serve as ground truth labels should be considered as *estimates* of the actual ground truth. The labels can be compared with the LIBS generated images as follows. On average, there are five LIBS measurements carried out per target. As mentioned in the previous section, the size of the LIBS pits is fixed and represented by $5 \times 5$ pixels, and the RMI image size is $1024 \times 1024$ pixels. LIBS measurements with high CaO abundance are denoted with white pixels, and those below 10 $wt\%$ are denoted with black pixels. On average, one can compare $5 \times 5 \times 5$ pixels out of $1024 \times 1024$, representing only $0.012\%$ of the RMI image. The number indicates the scale of the comparison. By comparing manually labeled masks with LIBS generated images in the area of the LIBS locations, we found $87\%$ of average overlap (here, ’overlap’ refers to the matching of pixel values). While checking the overlap along the pixel values, the white pixels showed $56\%$ match and the black pixels $97\%$. The high mismatch in labeling white pixels can be explained by the geological nature of the targets. As discussed above, elevated levels of CaO may be indicative of the presence of a light-toned vein. However, high CaO abundance can also be characteristic of plagioclase feldspar without light-toned veins^18,36^. Therefore, white pixels should be interpreted as a potential indication of light-toned veins, rather than definitive evidence of their presence. Additionally, light-toned veins on the target may be covered with a thin layer of dust that is removed during measurements, resulting in elevated calcium levels in the LIBS spectra but no vein visible yet in the RMI image taken before LIBS shots. One of the examples of a mismatch in the LIBS measurements and the visual inspection of the target is presented in Fig. 3A. At the circled point, no light-toned vein is visually detectable, yet the LIBS spectra show high calcium abundance, resulting in a mismatch between the two modalities. In Fig. 3B, another case of discrepancy is presented, introducing an additional factor contributing to the mismatch. Variations in lighting conditions affect the visual detection of diagenetic features in the RMI images. In this example, part of the target lies in shadow, making it difficult to determine whether light-toned veins are present within the shaded area.

In the case of black pixels in LIBS generated images, the mismatch between the RMI image and the calcium abundance may arise from the vein depth. As mentioned above, 30 LIBS measurements are performed at each point and subsequently averaged to determine the chemical abundance at that point. The mismatch may occur when a very thin vein visible in the RMI image does not extend deeply into the target, producing elevated calcium levels in the first few shots that quickly diminish and result in a low average calcium oxide abundance. Furthermore, LIBS sampling may mix vein and bedrock material, resulting in ambiguous abundance readings.

It is also important to consider that human error during pixel-level labeling may further contribute to discrepancies between LIBS-generated images and the ground truth labels. Altogether, these observations indicate that mismatches between LIBS measurements and the visual appearance of the target can arise from multiple factors, emphasizing that ground truth labels should be treated as estimates and that LIBS measurements alone may not be fully reliable for identifying light-toned veins. This highlights the potential of combining the two modalities to achieve better accuracy.

### Models

**Fig. 4:**
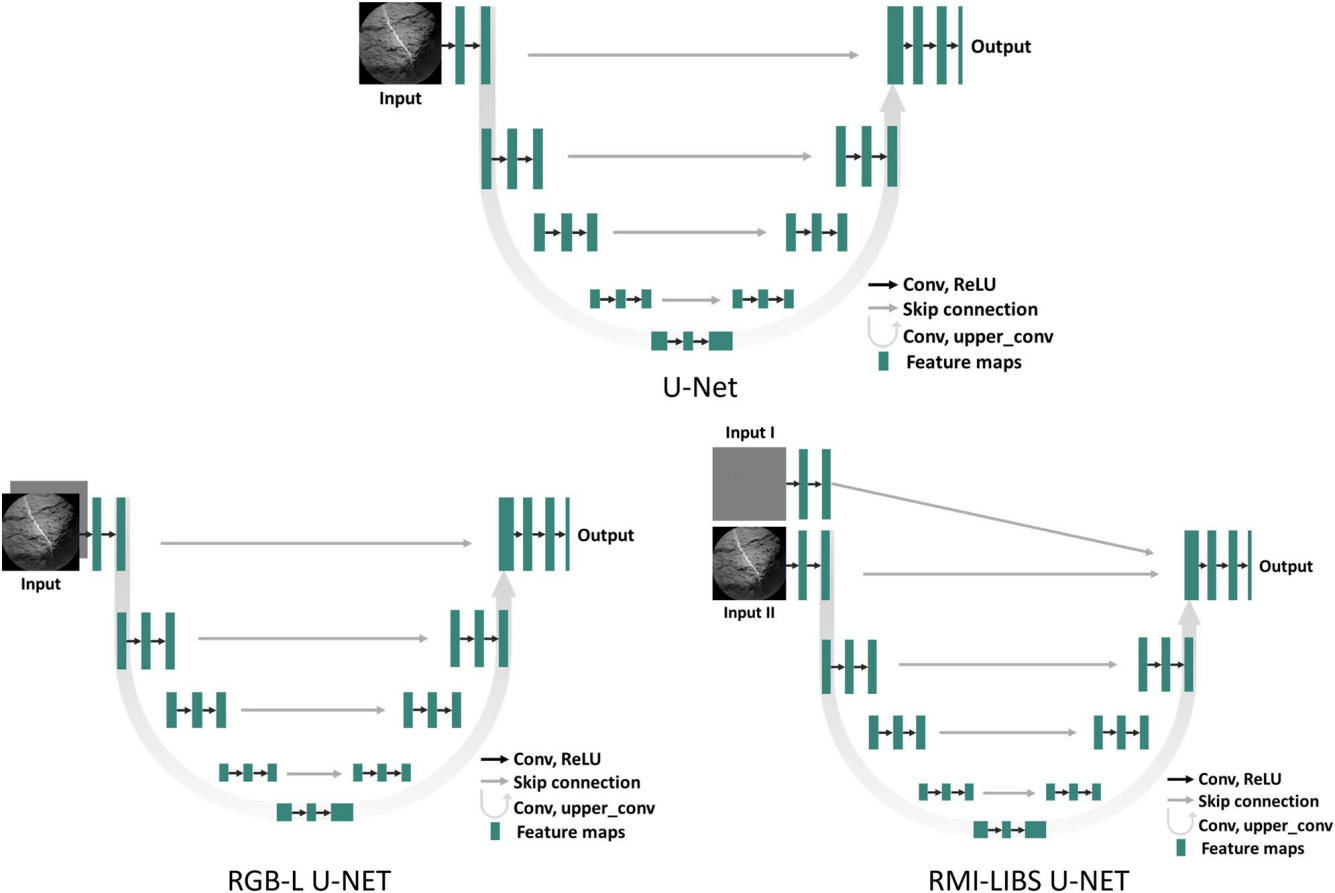
Three different settings of U-Net are shown. **U-Net** represents the classical architecture from the paper^25^. **RGB-L U-Net** introduces LIBS generated image as an additional channel to the input image. **RMI-LIBS U-Net** shows an architecture with two separate inputs: LIBS generated image and RMI image.

This work studies customizations to a classical U-Net^25^ based semantic segmentation model for processing ChemCam multimodal data. The U-Nets architecture is customized for the dataset described in the previous subsection in three ways: (i) weight initialization, (ii) addition of input channel, and (iii) designing a custom loss. The customizations of U-Net are shown in Fig. 4. The two methods presented on the bottom of the figure are specifically designed to incorporate LIBS data into the architecture and learning process. Modifications are made to a U-Net with a ResNet-34^37^ backbone. First, we have trained the classical U-Net architecture from scratch to semantically segment light-toned veins in the ChemCam targets. The detailed architecture is presented in the corresponding paper^25^. The weights are randomly initialized and training relied entirely on the RMI images. Although the images are acquired in grayscale, they are read as RGB images by duplicating the single channel across three identical channels. This approach introduces redundancy by providing the same information three times. However, since most deep learning models require three-channel inputs, we try to be consistent with the input values to ensure fair model performance comparisons. To train the model, different kinds of loss function are used, one binary cross-entropy (CE) and the second is the custom loss (CL), and the third is the combo loss (CL+FT), where FT stands for Focal-Tversky loss^38^.

As a baseline model, the classical U-Net architecture is selected and is referred to as Model 1 or simply as U-Net in Table 2. The second model is called CL-U-Net/ Model 2 and is a variant of U-Net that incorporates a custom loss function. The goal of CL is to force the model to pay attention to the LIBS locations by penalizing the pixel locations of the LIBS measurements in case the predictions did not match the Ca abundance. The custom loss function can be represented as follows:

1 $$\begin{aligned} L_{custom} = L_{ce}(T,P) + \eta L_{ce}(G_{x,y},P_{x,y}|x,y\in \text {LIBS}.) \end{aligned}$$

Here $L_{ce}$ stands for cross-entropy loss, *T* mask, *P* prediction, *G* LIBS generated images, *x*, *y* pixel locations, and $\eta$ is a weighting hyperparameter. The total loss is formed by adding an extra term to the regular cross-entropy loss, which is a cross-entropy loss between the LIBS generated images and the prediction, but only at the locations of the LIBS measurements.

For further customization of next models, pretrained U-Nets are considered, which are fine tuned on our dataset, i.e., a model is trained on a large dataset, and the learned embeddings are then used for a downstream task, which is segmenting light-toned veins in our case. For building a pretrained U-Net model, Segmentation Models PyTorch (SMP) library is employed^39^. It enables quick and easy implementation of different architectures for segmentation. For example, it provides an appropriate decoder once an encoder architecture has been chosen. The proposed models are pretrained on ImageNet dataset^40^. Model 3 is trained using the cross-entropy loss and is referred to as U-Net pretrained, whereas Model 4 employs a custom loss function and is referred to as CL-U-Net pretrained. Due to the fact that only a small fraction of pixels correspond to veins in the images, there is inherent class imbalance in the dataset. To address this, the custom loss function was combined with Focal-Tversky loss (FT)^38^ to form a combo loss for Model 5, referred to as Combo-U-Net pretrained. The combo loss is defined as follows:

2 $$\begin{aligned} L_{combo} = L_{custom} + FT \end{aligned}$$

This allows us to add LIBS information as well as handle class imbalance in the dataset. The following parameters were investigated $\alpha ,\beta \in {[0.1, 0.9]}$, $\gamma \in {[1,3]}$ for the Focal-Tversky loss. The $\alpha$ and $\beta$ parameters are used for weighting false negatives (FN) and false positives (FP), referring to the prediction of no veins in locations where veins are present and the prediction of veins in locations where none exist, respectively, and $\gamma$ is a focusing parameter for hard-to-classify pixels such as small regions or underrepresented classes. The parameter values $\alpha = 0.2$, $\beta = 0.8$, and $\gamma = 1.0$ were selected after hyperparameter tuning.

Next architectural changes are made to the basic U-Net in order to process LIBS generated images. The LIBS generated images are incorporated as a distinct input to the U-Net model in the following models: RGB-LIBS U-Net/Model 6 and RGB-LIBS CL-U-Net/ Model 7. We extract features from the LIBS generated images using the first block of encoder. We then concatenate the derived feature maps to the last block of the decoder using a residual connection (Fig. 4 bottom right). As one can observe, RMI images traverse the whole model, whereas LIBS generated images only go through the first few convolutional layers. The choice of architecture is driven by the nature of the images. RMI images are characterized by complex texture and require the extraction of more detailed features. In contrast, LIBS images, which have a low number of non-gray pixels, allow for efficient information summarization in only a few layers. Cross-entropy and custom loss are used to train the proposed models: RGB-LIBS U-Net/ Model 6 and RGB-LIBS CL-U-Net/ Model 7 respectively. These models are initialized with random weights.

Another data fusion approach can be implemented by adding the LIBS image as an additional channel to the RMI image. For example, if there are three channels, such as RGB, the LIBS-generated image can be a fourth channel. The motivation is to add additional information for LIBS locations in the input data and propagate it throughout the network. Figure 4 bottom left shows the simplified schematics of the architecture. This model is referred as RGB-L U-Net/ Model 8.

For comparison with other state-of-the-art models, transformer-based model DINOv2^26^ is used. Also, a decision forest based model, TextureCam, earlier developed for such segmentation presented in^27^ is used for comparison. The starting learning rate for deep learning models is 0.0001, which is reduced by the learning rate scheduler *ReduceLROnPlateau* with a reduction factor of 0.9 and patience of 10. Training is carried out until overfit is observed. The value of $\eta$ in (1) after hyperparameter tuning is set to 1. The used optimizer is *Adam*, input Image size $256 \times 256$. Hyperparameters are summarized in Table 1.

**Table 1:** Hyperparameters for the deep learning models. CE denotes cross entropy loss, while CL refers to the custom loss function. CL+FT represents a combined loss of custom loss and Focal-Tversky loss.

#	Models	LIBS	Batch size	Loss
1	U-Net	–	16	CE
2	CL-U-Net	+	8	CL
3	U-Net pretrained	–	16	CE
4	CL-U-Net pretrained	+	16	CL
5	Combo-U-Net pretrained	+	16	CL+FT
6	RMI-LIBS U-Net	+	16	CE
7	RMI-LIBS CL-U-Net	+	16	CL
8	RGB-L U-Net	+	16	CE
9	DINOv2	–	8	CE

### Risk control

The integration of deep learning across diverse domains has created the need to quantify the uncertainty of model predictions and establish domain-appropriate risk control mechanisms. For example, in medical imaging, a false negative, such as failing to identify a tumor region, carries far greater consequences than a false positive, which typically results only in further diagnostic testing. Consequently, models are calibrated to prioritize a specific type of error depending on the application^41^. We have employed such a calibration method to provide a statistical guarantee on the predictive uncertainty of our model. We utilize the Learn-Then-Test (LTT)^42^ method to post process the outputs of the model without retraining it. LTT generates a $(\epsilon , \delta )$ risk control prediction (RCP) that bounds the user defined risk, *R* as, $\textbf{P}(R(T_{\hat{\lambda }})\le \epsilon ) \ge 1 - \delta$. The risk tolerance $\epsilon$ and the error level $\delta$ are chosen by the user. $T_{\lambda }$ is a class of functions parameterized by $\lambda \in \Lambda$ that threshold the output of the model, e.g. sigmoid outputs in our case, at $\lambda$, thus, indirectly parameterizing the risk by generating different segmentation maps. The aim of LTT is then to find $\hat{\lambda }$ that generates an $(\epsilon , \delta )$ RCP. Each $\lambda _i \in \Lambda$ is associated with a null hypothesis $R(T_{\lambda _i}) > \epsilon$, and a finite sample p-value is computed for each hypothesis. A family-wise error rate control (FWER) algorithm then generates a set of $\lambda$ where the risk is controlled. Any $\hat{\lambda }$ in this set will produce the required RCP. This is a post-processing technique and does not require retraining of the model. It sits as a wrapper on top of the segmentation model. The implementation of LTT from the MAPIE (Model Agnostic Prediction Interval Estimator)^43^ library is used. The calibration set was the same as the validation set. Here, false discovery rate (FDR) (3) is chosen as the risk and we choose the $\hat{\lambda }$ that maximizes the precision. Bonferroni correction is used to control the FWER^42^. Here, FP is the false positives as described in the FT loss and TP refers to true positives, the prediction of veins in locations where they are present, respectively.

3 $$\begin{aligned} {\textrm{FDR}} = 1-Precision = \frac{\textrm{FP}}{\textrm{TP} + \textrm{FP}} \end{aligned}$$

In the simulations $\epsilon = 0.1 \text { and } \delta = 0.1$, meaning that the calibrated model’s predictions are constrained to have an FDR of no more than 10%. This choice of FDR, high confidence in true positives, was motivated by potential applications involving the investigation of chemical composition of light-toned veins using LIBS data. Since false positives, the incorrect prediction of vein presence, can lead to erroneous LIBS data comparisons, FDR was considered to be the appropriate metric.

## Results

This section summarizes the performance of the customized U-Net models with various settings for semantic segmentation of ChemCam data. Across model variants, the impact of incorporating LIBS data to the training set as an input or as an additional parameter in the loss function is investigated. The cross entropy loss is selected as a baseline objective function and the obtained results are compared to the results obtained by a custom loss with the LIBS information and Focal-Tversky (FT) loss^38^. The U-Net architecture-based models are subsequently compared with the transformer-based DINOv2 and the random forest-based TextureCam algorithm^26,27^. All models are trained and validated on the proposed dataset. Alongside numerical evaluation of the performance, visual inspection of the best-performing models is presented, and challenging cases are explored.

### Model accuracy

Table 2 shows the performance of the considered models and their setups. The column LIBS indicates whether LIBS generated images have been incorporated into the model. Column Loss specifies which loss function was used for optimization. Here, CE denotes the cross-entropy loss, CL to custom loss as in (1), and CL+FT is the combined loss as in (2). The last two columns present the performance metrics in percentages. It should be noted that, for both mean Intersection over Union (mIoU) and F1 score, values of 70% or higher (IoU) and 80% or higher (F1) are generally regarded as indicative of good semantic segmentation performance^44–46^.

The results show that the models based on U-Net architecture demonstrate high accuracy, with mIoU values exceeding 73% and F1 scores above 84%. According to both metrics, the best performing models (in bold), U-Net/Model 2 and U-Net pretrained/Model 4, employ the custom loss function. Even though the LIBS points are limited in number, the penalty they introduce during training guides the model to adhere to the spectral information and provide LIBS plausible segmentation. The next best performing model is U-Net pretrained with cross-entropy loss/Model 3. The model no longer has an additional penalty introduced by LIBS points. Based on the previous cases, increasing the complexity of the loss function appears to improve model performance; however, this improvement is not observed when combining cross-entropy with Focal-Tversky loss in Model 5, where a slight performance decline is observed relative to Model 4. Overall, the weakest U-Net–based models are those without pretrained weights and without a custom loss function. Incorporating LIBS-generated images as an additional input or channel does not lead to improved model accuracy; instead, the most notable performance gain is achieved when LIBS information is integrated through the loss function.

Moving towards other types of architectures, the transformer-based DINOv2 shows lower performance than simple U-Net/ Model 1. The only model it outperforms is the RGB-L U-Net/ Model 8. For comparison, we also evaluate TextureCam, a random-forest-based classifier^27^. This decision-tree-based model, tested for the semantic segmentation of light-toned veins, performs weaker than all deep learning approaches. In general, these results present the advantage of convolutional neural network-based architectures over both transformer-based models and classical machine learning methods for the task of semantic segmentation of light-toned veins using multi-modal RMI and LIBS data.

**Table 2:** Model performance across architectures, loss functions, and LIBS configurations (“+” indicates inclusion of LIBS data). Highlighted are the best performing models. The table is divided vertically into two groups, separating U-Net–based models from all other architectures. CE, cross-entropy; CL, custom loss; FT, Focal–Tversky.

#	Models	LIBS	Loss	mIoU %	Mean F1 %
1	U-Net	–	CE	76.1	86.5
2	**CL-U-Net**	+	**CL**	**79.7**	**88.7**
3	U-Net pretrained	–	CE	78.6	88.0
4	**CL-U-Net pretrained**	+	**CL**	**80.1**	**88.9**
5	Combo-U-Net pretrained	+	CL+FT	78.4	87.9
6	RMI-LIBS U-Net	+	CE	77.0	87.0
7	RMI-LIBS CL-U-Net	+	CL	77.8	87.5
8	RGB-L U-Net	+	CE	73.6	84.8
9	DINOv2	–	CE	75.6	83.3
10	TextureCam	–	–	57.9	66.6

### Visual inspection of predictions

**Fig. 5:**
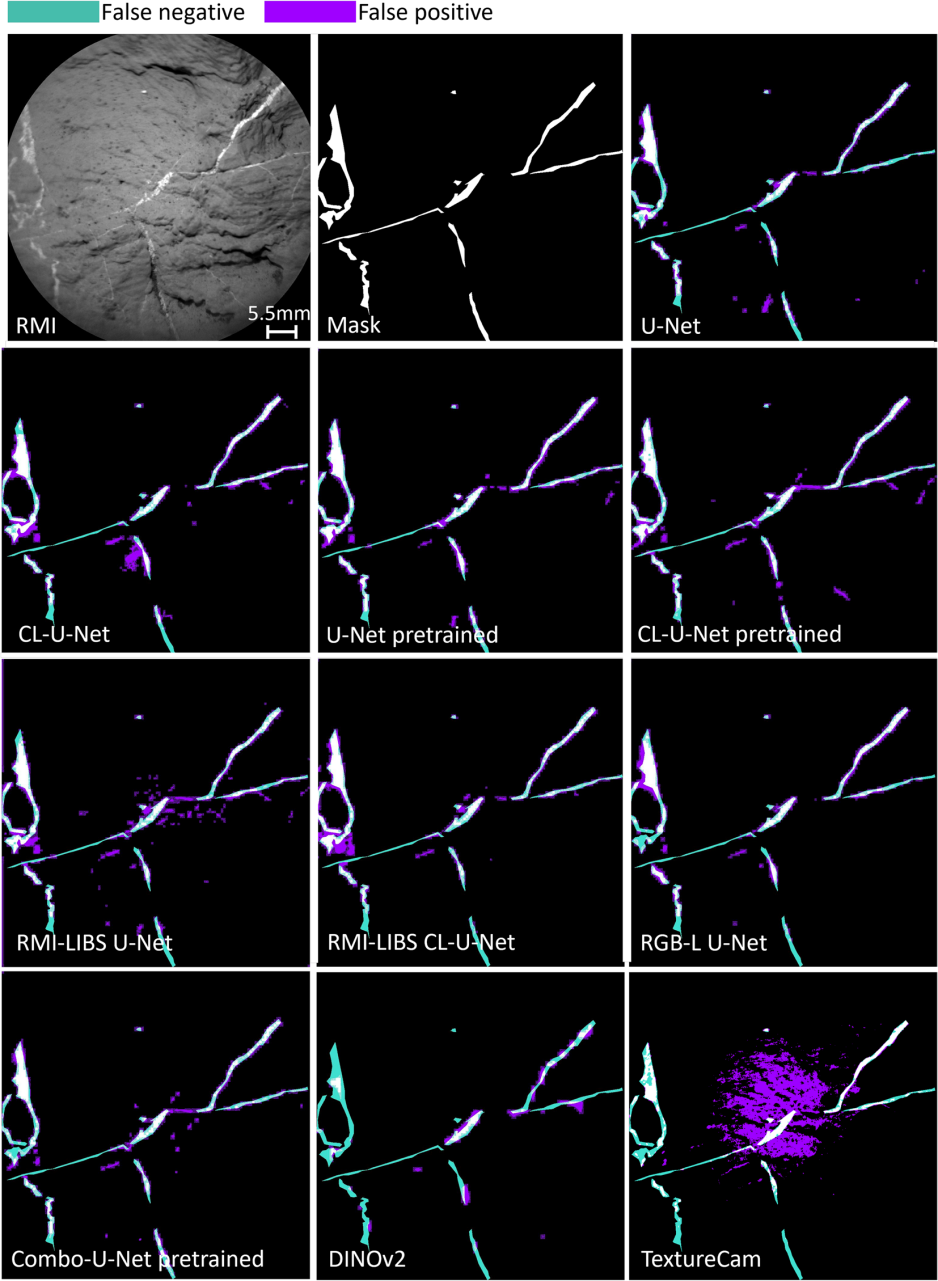
The first image in the upper left corner is AEGIS target from sol 2784. The neighboring image on the right is a manually developed mask. The rest of the images are predictions of the implemented models, with highlighted erroneous pixels. Green represents false negatives, and purple shows false positives. The target represents one of the samples from the test set.

In this subsection, the predictions of all models are explored visually. In the first case, one of the targets is selected from the test set. The AEGIS target from sol 2784 (AEGIS_post_2784a, ccam03783), shown in Fig. 5, has a layered texture and thin fractures filled with calcium sulfate. Having a textured rather than a smooth surface makes the target an interesting test sample for all the models. Furthermore, the RMI image displays blurring along the left edge, which introduces noise into the input image. Figure 5 shows the RMI image of the target, mask, and predictions from all the models previously discussed. The difference between the predictions of most models is not particularly significant. Furthermore, predictions of certain U-Net based models, including U-Net, U-Net pretrained with and without custom loss, predict more thin veins than the mask displays. By comparing these predictions to the RMI image, one can see that the models may be more sensitive to small details than the manual annotations. On the other hand, DINOv2 underfits and does not recognize thin veins, resulting in high false negative values presented in green. TextureCam overfits highly in the center of the target, shown in a high concentration of false positives in purple. The orientation of the purple segments follows the rock layering, indicating that the model is misclassifying the layered textures as light-toned veins.

**Fig. 6:**
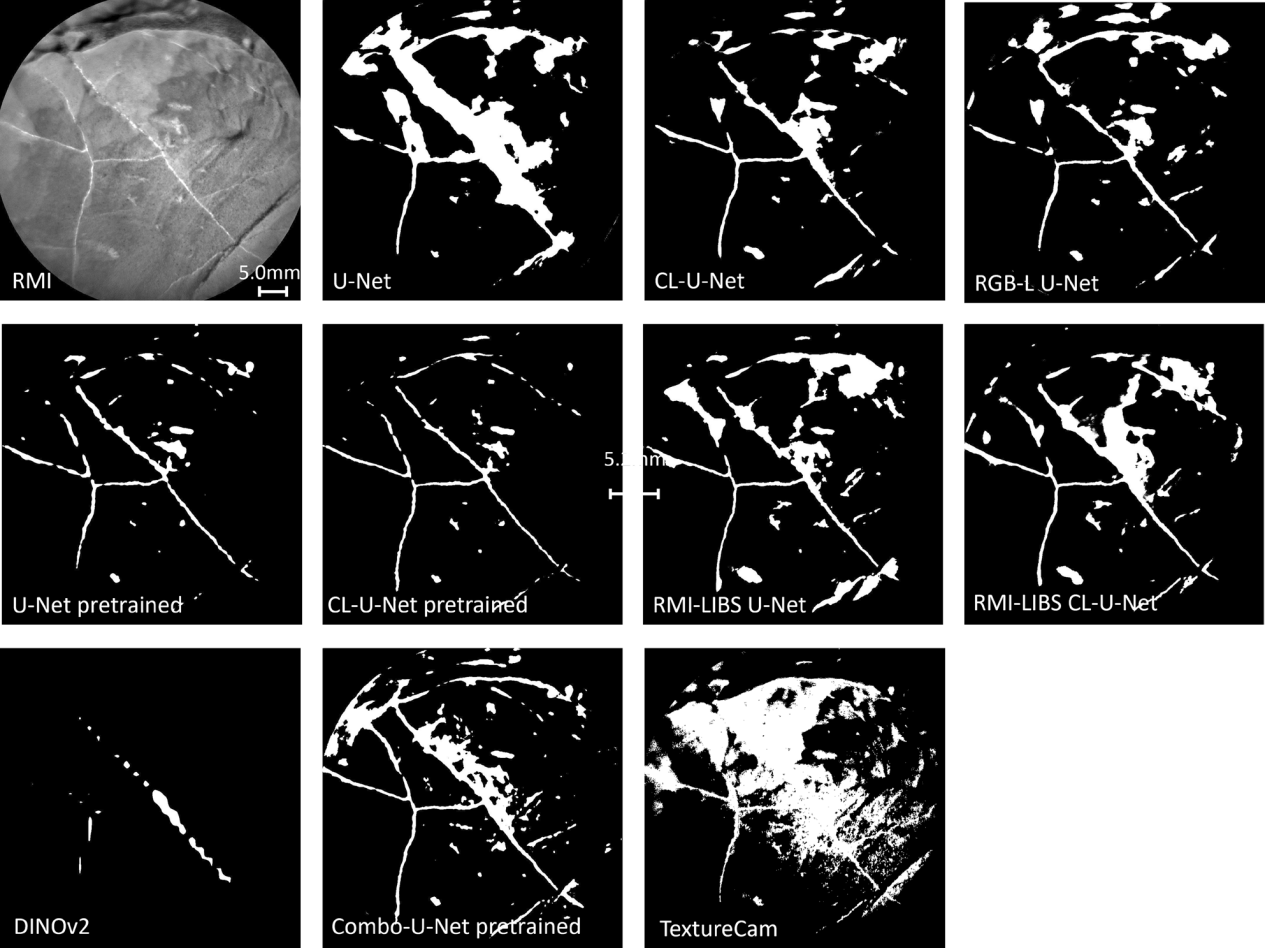
First image in the upper left corner shows target Holyrood_ccam sol 1906. The target represents one of the challenging images for light-toned vein identification due to its shiny surface and the occurrence of the bedrock’s edge. The rest of the images show light-toned vein predictions of the listed models. .

In order to better differentiate the performance of models, a more challenging sample is selected from outside the labeled dataset. Figure 6 shows the target Holyrood_ccam from sol 1906. The lighting conditions cause the target to appear shiny and reduce the contrast between the light-toned veins and the rest of the target. In addition, the RMI image shows the edge of the bedrock displaying shiny spots caused by light reflection, which can be mistaken for a light-toned vein. Given the described challenges, the target was not included in the training set and therefore no manually labeled mask exists for this image. The difference in the predictions among the models is more obvious for this target compared to the previous one in Fig. 5. U-Net/Model 1 is overfitting and predicting much larger veins. Adding custom loss to the U-Net model has been shown to improve the prediction accuracy of veins (Table  2). However, the model continues to experience challenges with shiny reflections in the middle. The most accurate model is Model 4, CL-U-Net pretrained, where initializing with pretrained weights and addition of a custom loss enhances the model’s performance. Adding LIBS generated images as an input or an additional channel improves the initial U-Net model/Model 1. However, all U-Net based models continue to encounter challenges on the edge of the bedrock and with shiny reflections. Although challenging lighting conditions are no longer an issue for DINOv2, the model remains unable to detect thin veins, and the vein it identifies near the center is predicted to be thicker than it actually is. Moving to the TextureCam, the model has difficulty with the lighting and the texture of the target. It overpredicts veins and again produces false positives aligned with the rock’s layering.

**Fig. 7:**
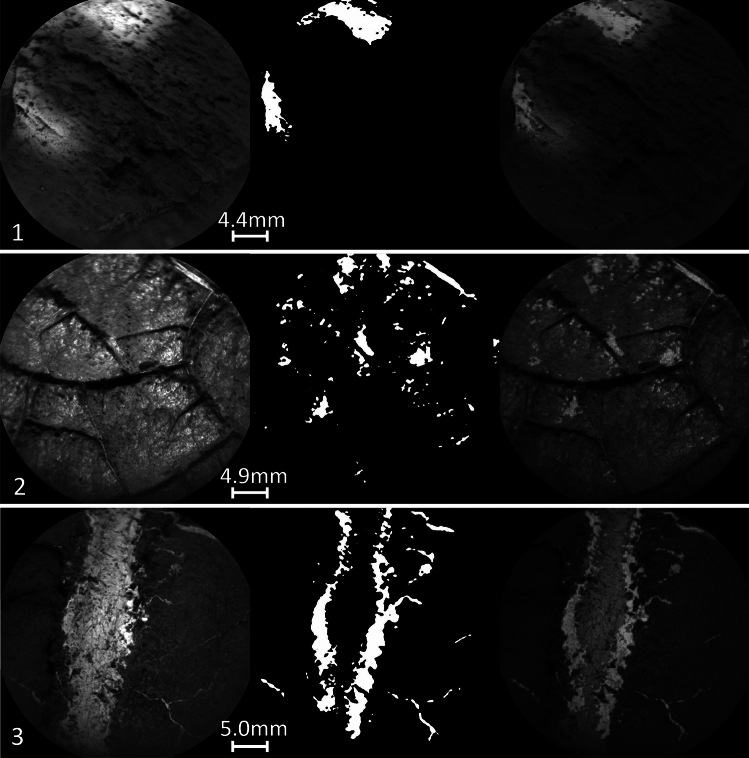
Three rows display different challenging targets and predictions of light-toned veins in them. The first column shows RMI images fed to U-Net pretrained with custom loss/ Model 4. The second column shows predictions of the samples, and the third column is an overlap of the RMI images with the corresponding predictions. (1) Target Beinn-an-dudhaich sol 2737 displays light-toned veins in the lower right corner, which are challenging to identify due to shadowing. The model also faces difficulties detecting the veins and misidentifies sunlit areas of the bedrock as veins (center top). (2) Target Northeast-harbor sol 1566 is a challenging target due to its shiny surface, ridges, and thin light-toned veins. (3) Target Onganja sol 1363 has a very thick light-toned vein in the center..

The limitations of the best performing model, U-Net pretrained/ Model 4 with custom loss, are explored by further inspections of challenging targets. By feeding the model with targets predicted by the texture classifier to contain light-toned veins along the traverse, the following challenging conditions were identified: (1) Due to lighting conditions, the model is unable to detect light-toned veins in shaded targets. In addition, sunlit areas might be misclassified as light-toned veins. (2) As indicated previously, the model’s ability to accurately predict shiny targets remains a challenge. (3) Extremely thick or very thin light-toned veins are difficult to detect precisely. In Fig. 7, examples of targets corresponding to challenging conditions are provided.

### Calibration results

**Fig. 8:**
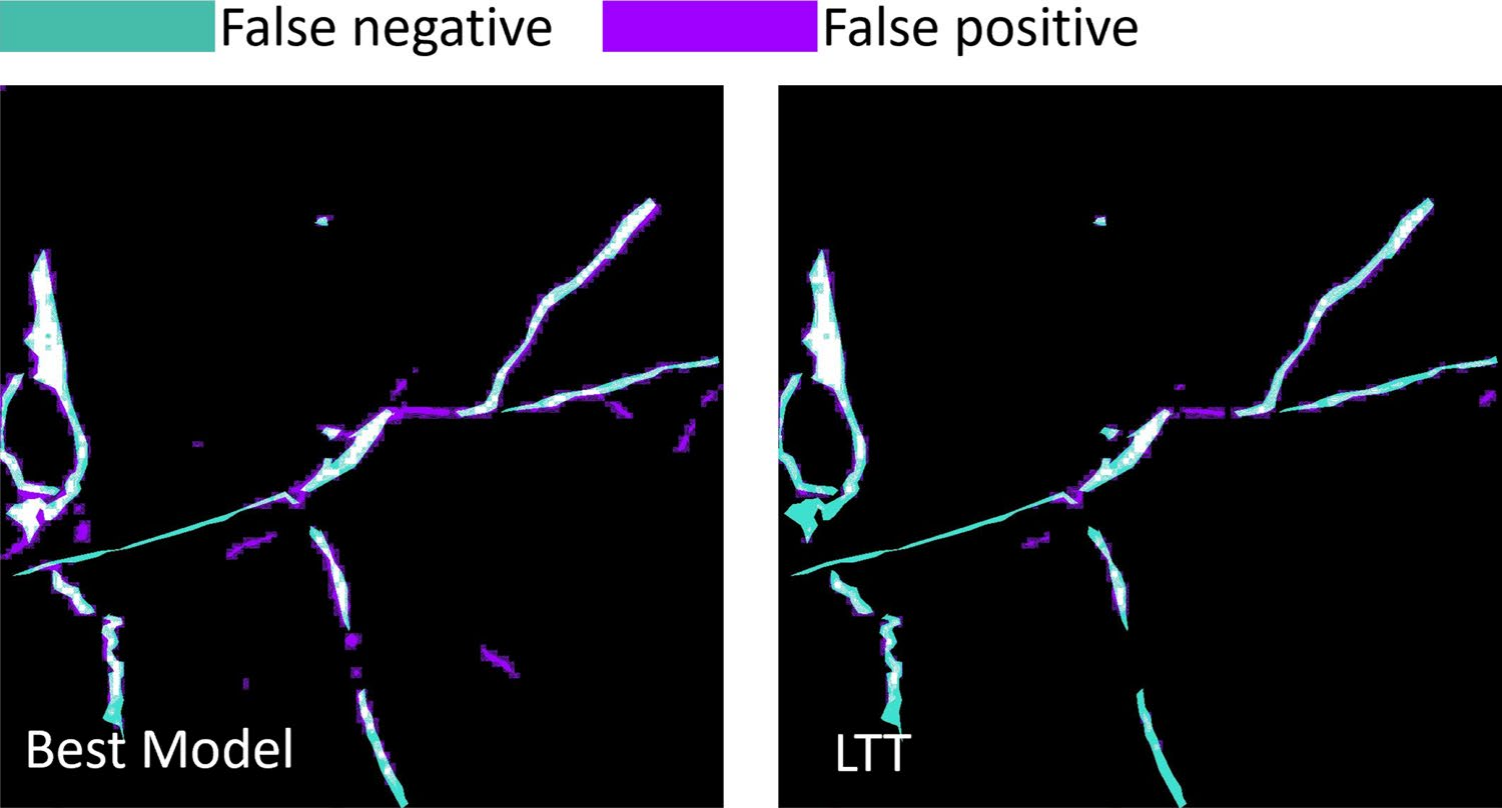
Left: prediction from the U-Net pretrained with the custom loss. Right: prediction from the same model after LTT calibration. Compared to the initial prediction on the left, LTT calibration reduces the false positive rate (highlighted in purple), at the cost of an increase in false negatives (highlighted in turquoise).

LTT method was applied to the pretrained U-Net model with custom loss/Model 4. As mentioned above, 10% was set as a threshold for FDR. The test set before calibrating the model showed 15% FDR, and after calibration it showed 7%. Figure 8 shows a test sample before calibration on the left and after LTT on the right. The false positive rate in purple decreases on the right. Although false negatives increase, the calibrated prediction meets the set threshold for FDR.

## Discussion

The original U-Net architecture/ Model 1 is selected as the baseline model in this work^25^. This baseline U-Net is customized for multimodal ChemCam data. The experimental setup of the architecture involves adding the LIBS generated image to the model, the custom loss function, and the combined loss function of Focal-Tversky and custom loss is analyzed by comparing accuracy metrics as well as by visual inspection. Finally, U-Net based models are compared to the transformer-based DINOv2 and TextureCam, a decision tree model designed for the rover to detect light-toned veins.

We further break down the results section by discussing the challenges that the model faces. Understanding these challenges requires considering the diversity of the data. ChemCam captured more than 4000 targets of at least 17 different diagenetic features^7,22^. Since deep learning models perform well on large sets of similar targets (e.g., 1000 samples per class), the limited size and strong heterogeneity of the ChemCam RMI samples make the dataset particularly challenging^47^. For example, light-toned veins can appear in both smooth as well as in nodular rocks, and during training, the model may not encounter all possible textures or may develop a bias toward certain types, which can confuse the model and negatively affect overall performance. The difficulty is compounded by the challenges of pixel-level labeling of thin diagenetic features, resulting in a small training set with labels that inevitably contain some errors. All these factors lead to a generalization problem, which means that the model demonstrates high accuracy (mIoU > 70%) on the training data, but can underperform on certain unseen data. To address this challenge, standard data augmentation techniques, such as image tiling and geometric augmentations, are implemented. In addition, the residual connections of the U-Net architecture allow the model to propagate low and high-level features throughout the whole network, aiding in generalization even with limited dataset sizes.

According to the performance metrics in Table 2, the baseline model/ Model 1 already demonstrates strong performance. The visual inspection in Fig. 5 also suggests using the baseline model with a reasonable degree of confidence. Comparison of the mask to U-Net predictions shows that the false negatives in green are slightly dominating the false positives in purple. According to this sample, one might think that the model is underfitting, but the U-Net/ Model 1 prediction of the challenging sample in Fig. 7 shows a highly overfitting prediction. The model is unable to distinguish between light-toned veins and shiny parts of the rock. Other prominent false positive pixels are on the edge of the rock. The model is confused with the sample in which the surface of the rock does not fully cover the field of view of the RMI. At the top of the RMI image, one might observe soil with pebbles, which the model also misinterprets as veins. Overall U-Net/ Model 1 performs well on samples where veins are clearly visible, and the method can be used for fast and efficient retrieval of light-toned veins’ locations in RMI images with a high level of performance.

Considering only Fig. 5, it is hard to evaluate the changes in the performance of U-Net model caused by adding a custom loss function or LIBS generated images. On the other hand, Fig. 6 displays the improvements in predictions across models. Although the number and size of the LIBS pits is small compared to the whole RMI image, adding a term to the loss function forces the model to train more and learn more detailed features of the light-toned veins as can be seen in Fig. 6.

The custom loss function did not improve the model with a separate input of LIBS generated image/ Model 7. RMI-LIBS performs better than U-Net/Model 1, but is not able to outperform the U-Net model with custom loss/ Model 2. The reason might be the increasing number of parameters. By adding a LIBS generated image, the number of trainable parameters increases, but the size of the training set remains the same. This can lead to weak generalization of the model.

The same applies to adding a LIBS-generated image as an additional input channel/ Model 8, which increases the number of trainable parameters. The best performing models are U-Net with custom loss/Model 2, pretrained U-Net/ Model 3, and the same model with a custom loss function/Model 4. Introducing the learned embedding from a much larger dataset allows the model to identify more detailed features of fractures filled with calcium sulfate, rather than just detecting changes in tones of the target when having a light-toned vein. In Fig. 5, Model 3 shows slight overfitting, yet successfully identifies the edge of the rock and reflections from light-toned veins. Adding a custom loss function/Model 4 improves mIoU, as well as mean F1 scores. The prediction in the figure also shows an increase in confidence of detected veins as it displays smoother veins compared to dashed ones in the pretrained U-Net/ Model 3. In an attempt to improve the model’s capabilities and address the class imbalance, Focal-Tversky loss is added to the custom loss/Model 5. After tuning the model across more than 100 hyperparameters, the best performance was achieved with $\alpha =0.2, \beta =0.8, \gamma =1.0$. This corresponds to assigning a higher weight to false positives and reducing the Focal–Tversky loss to the Tversky index, since $\gamma =1.0$. The performance metrics, as well as visual inspection of the challenging sample in Fig. 6, show that although Model 5 achieves high accuracy scores, it exhibits a high false positive rate in the challenging sample. This suggests that increasing the complexity of the loss function further can lead to overfitting.

Considering the remaining models, the transformer-based DINOv2/Model 9 achieves adequate accuracy but still underperforms most U-Net-based architectures. Visual inspection indicates that the model handles challenging lighting conditions reasonably well, producing few false positives. However, it continues to struggle with the detection of thin veins. This can be explained by the nature of the foundation model. Transformers, including DINOv2, often struggle with very thin or small structures, such as thin veins, because these require high-resolution local detail and spatial precision, which may be lost in the patch-based attention mechanism^48^. The final model is TextureCam, based on decision trees. It misclassifies layers on rocks as light-toned veins. Due to their architecture, decision trees rely on local and shallow splits, which prevent them from effectively capturing the distinction between high-level features, such as rock layers, and fine-scale details, such as thin light-toned veins. In contrast, U-Net is specifically designed to learn and propagate low-level and high-level features throughout the network via its encoder–decoder structure and skip connections, allowing precise segmentation at the pixel-level.

In general, the study revealed that the pretrained U-Net model with a custom loss function outperformed the other presented models. This motivates us to investigate the model further and identify RMI images that cause the model confusion. By running the best performing segmentation model/ Model 4, the following challenging conditions were identified, presented in Fig. 7. The first is the illumination conditions. The strong contrast created by the simultaneous presence of sunlit and shadowed areas on the rocks can be mistaken for light-toned veins. For humans, it is easy to recognize that these light spots are due to sunlight, but the model is confused by the lack of such samples in the training set. The second image shows a target with a shiny surface and raised ridges that does not seem to be made of calcium sulfate. This case presents similarities to the challenging target presented in Fig. 6 but is covered with more shiny spots and thin light-toned veins. The third row shows a very thick vein. Surprisingly, the model is able to detect fine light-toned veins on the edges but misses the very thick major vein in the center. Again, this is an example of how the training set influences the performance of the model. Such thick veins are not frequently observed in the training set. Therefore, during training, the model develops embeddings that better describe thin light-toned veins than thick ones. One might assume that the first and the third examples can be dealt with by refining the training set, but in general such samples are not common. The thin veins are observed more frequently than thick veins, leading to an imbalance between these two types of light-toned veins. Since foundation models tend to be robust to challenging lighting conditions and are good at detecting dense objects, further exploration of these architectures may lead to performance improvements. Although the second case, where the shiny surface interferes with light-toned veins and even the trained human eye has difficulty determining where the vein begins and ends. In such scenarios, it is helpful to have some performance guarantees on the model output. The Learn-Then-Test (LTT) framework^42^ can produce risk control predictions such that the risk at the output of the model after LTT is within a user-defined tolerance. The procedure is further described in the “Methods” section, but one calibrates the model outputs to the expected scene. This allows a flexible method to provide statistical risk controlled guarantees about the model outputs. This is particularly advantageous in scenarios where retraining is impractical or resource-constrained such as onboard planetary rover operations. In our case, the threshold was set to have high confidence in the predicted locations of light veins. Compared to the prediction of the uncalibrated model, the LTT method provided a guaranty of being consistent with the specified risk threshold, the FDR became 7% at the cost of higher false negatives. Although application-specific calibration may reduce recall, the accompanying increase in precision and the ability to specify desired error rates make LTT a valuable tool for mission-critical and scientific applications.

## Conclusion

In this work, we introduce a pixel-level labeled, multimodal dataset of *Curiosity* rover’s ChemCam observations in Gale Crater, Mars and study a semantic segmentation model of light-toned veins in RMI images of LIBS targets. The task is to efficiently identify light-toned veins with predefined confidence scores that support further geological analysis. The standard practice for identifying light-toned veins, i.e., fractures filled with calcium sulfate, is to manually go through every image and visually inspect them^10,11^. By providing the dataset and the customized U-Net–based models, the work allows automated, confidence-controlled detection of light-toned veins in ChemCam RMI images. The predictions of the best performing model can serve as a basis for further characterization and study of light-toned veins along the rover’s traverse, including analyses of their chemical composition and morphological properties such as vein width.

Due to the lack of labeled data, a multimodal dataset was developed that involved manually annotating masks displaying light-toned veins in RMI images, along with the images containing chemical information based on LIBS spectra. We then implemented and evaluated several customization to the U-Net to identify the best-performing architecture for this task. The experiments included adding LIBS information to the input, testing with pretrained weights, and implementing a custom loss function. To benchmark our approach, the proposed eight models were compared with the state-of-the-art transformer based model, DINOv2 as well as a random forest based classifier. Among all the evaluated models, CL-U-Net pretrained/Model 4, with pretrained weights and a custom loss function incorporating LIBS information, showed the best performance with the mIoU of 80.1%, compared to 75.6% for DINOv2 and 57.9% for TextureCam. Furthermore, visual inspections of the validation data showed the model’s sensitivity to fine-scaled veins, which were not easily labeled by humans.

The model’s limitations were explored by inspecting challenging samples. While some factors, like shadows and lighting variability, are unavoidable, the model’s performance remains convincing. Further improvements are expected with the investigation of foundation models, integration of complementary data sources such as Mastcam^49^ images, and use of the full set of available chemical information. Moreover, future studies should explore noise-robust learning strategies and incorporate explainability-guided modeling approaches to enhance model robustness and interpretability^50,51^. In its current form, the model enables an efficient combination of different data modalities by using information from RMI and LIBS modalities, improving mIoU from 76.1 to 79.7% for U-Net, and from 78.6 to 80.1% for U-Net pretrained. Additionally, risk control enables statistically guaranteed predictions with respect to an application specific risk. We consider FDR as the risk and use the LTT framework to produce risk controlled segmentation maps. Overall, the model not only improves current approaches for detecting light-toned veins, but also motivates the future work of extending semantic segmentation to other texture classes and composition, such as nodules, and further exploration of deep learning models for Martian in-situ data.

## Supplementary Information

Supplementary Information.

## Data Availability

The dataset and code developed for this study are owned by DLR. Access can be provided by the authors upon reasonable request. Images and composition data used to create dataset in this work are available on the Planetary Data System.
