# Supplementary material for: Semantic segmentation of light-toned veins in multimodal ChemCam data
Source: Sci Rep. 2026 Apr 9;16:12052. doi: 10.1038/s41598-026-47207-0 (PMC13068912; doi:10.1038/s41598-026-47207-0)
Supplement: Supplementary file 1 — Supplementary Information. [file 41598_2026_47207_MOESM1_ESM.pdf]

# Supplementary materials in support of the paper: Semantic Segmentation of Light-Toned Veins in Multimodal ChemCam Data

**Ana Lomashvili[1,3*], Kristin Rammelkamp[1*], Protim Bhattacharjee[1], Olivier Gasnault[2], Elise Clavé[1], Christoph H. Egerland[1], Susanne Schröder[1], Travis S.J. Gabriel[4], Ari Essunfeld[5], Stéphane Le Mouélic[6], and Begüm Demir[3]**

[1]German Aerospace Center (DLR), Institute of Space Research, Berlin, Germany.
[2]Institut de Recherche en Astrophysique et Planétologie IRAP, Université de Toulouse, CNRS, CNES, Toulouse, France
[3]BIFOLD and TU Berlin, Berlin, Germany.
[4]U.S. Geological Survey, Astrogeology Science Center, Flagstaff, AZ
[5]Los Alamos National Laboratory, Los Alamos, 87545, New Mexico, USA
[6]Laboratoire de Planétologie et Géosciences, CNRS UMR 6112, Nantes Université, Univ Angers, Le Mans Université, 44000 Nantes, France.
[*]Address correspondence to: ana.lomashvili@campus.tu-berlin.de or Kristin.Rammelkamp@dlr.de

The ChemCam RMI dataset presents several challenges for sensitivity analysis of automated detection of light-toned veins. The distance between the camera and the target varies across observations, and the geometric configuration of the target relative to the camera is not constant. Furthermore, images were acquired under varying illumination conditions and spatial resolutions. These factors make sensitivity analyses of the model's vein detection performance difficult, particularly with respect to vein size, morphology, and lighting conditions. Therefore, we evaluate the best performing model's, CL-U-Net pretrained/ Model 4, performance under controlled simulated conditions.

In Supplementary Fig. 1, we show the variation in segmentation performance, measured by mean Intersection over Union (mIoU), as a function of synthetic resolution degradation applied to the test images. Image blurring is introduced by downsampling the original RMI images using OpenCV and subsequently upsampling them back to their original dimensions, thereby preserving image size while progressively degrading spatial detail. The best-performing model achieves an mIoU of 80.1% on the original images. As the level of degradation increases, the segmentation performance progressively decreases, eventually falling below an acceptable performance threshold (mIoU < 70%).

In Supplementary Fig. 2, we present a sensitivity analysis of the model with respect to changes in lighting conditions. Different illumination levels are simulated using the OpenCV cv2.convertScaleAbs function by varying the scaling parameter $\alpha$, which controls the image contrast. Under strongly overexposed conditions ( $\alpha = 2$), where the contrast between light-toned veins and the surrounding rock is significantly reduced and vein boundaries become difficult to identify even by visual inspection, the model performance decreases substantially, reaching an mIoU of approximately 50%. As the contrast between the veins and the background increases, the segmentation performance improves accordingly. Although the model's accuracy also decreases under low-light conditions ($\alpha < 1$), the reduction in performance is less pronounced because the contrast between veins and the background remains partially preserved.

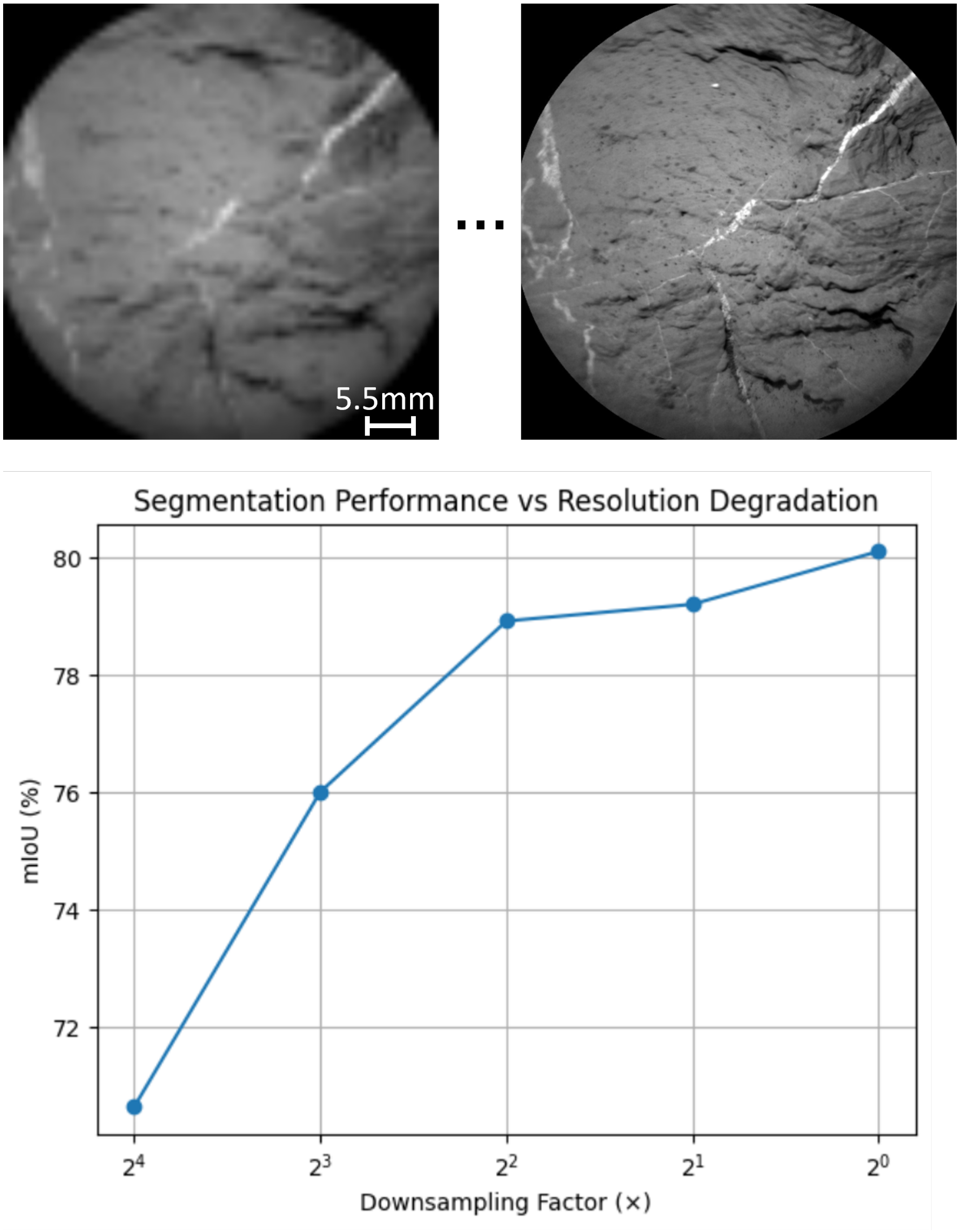


**Figure 1.** *Sensitivity of segmentation performance to resolution degradation. Downsampling is implemented using OpenCV: the original RMI is first resized by a factor x and subsequently upsampled back to its original dimensions, resulting in images of the same size but with increasing blur for stronger downsampling. Top left: Example of an RMI downsampled with a factor of* $2^4$*. Top right: Example of an RMI with a downsampling factor of* $2^0$*, corresponding to the original RMI. The Bottom panel shows the mean Intersection over Union (mIoU) of the best-performing model as a function of the downsampling factor.*

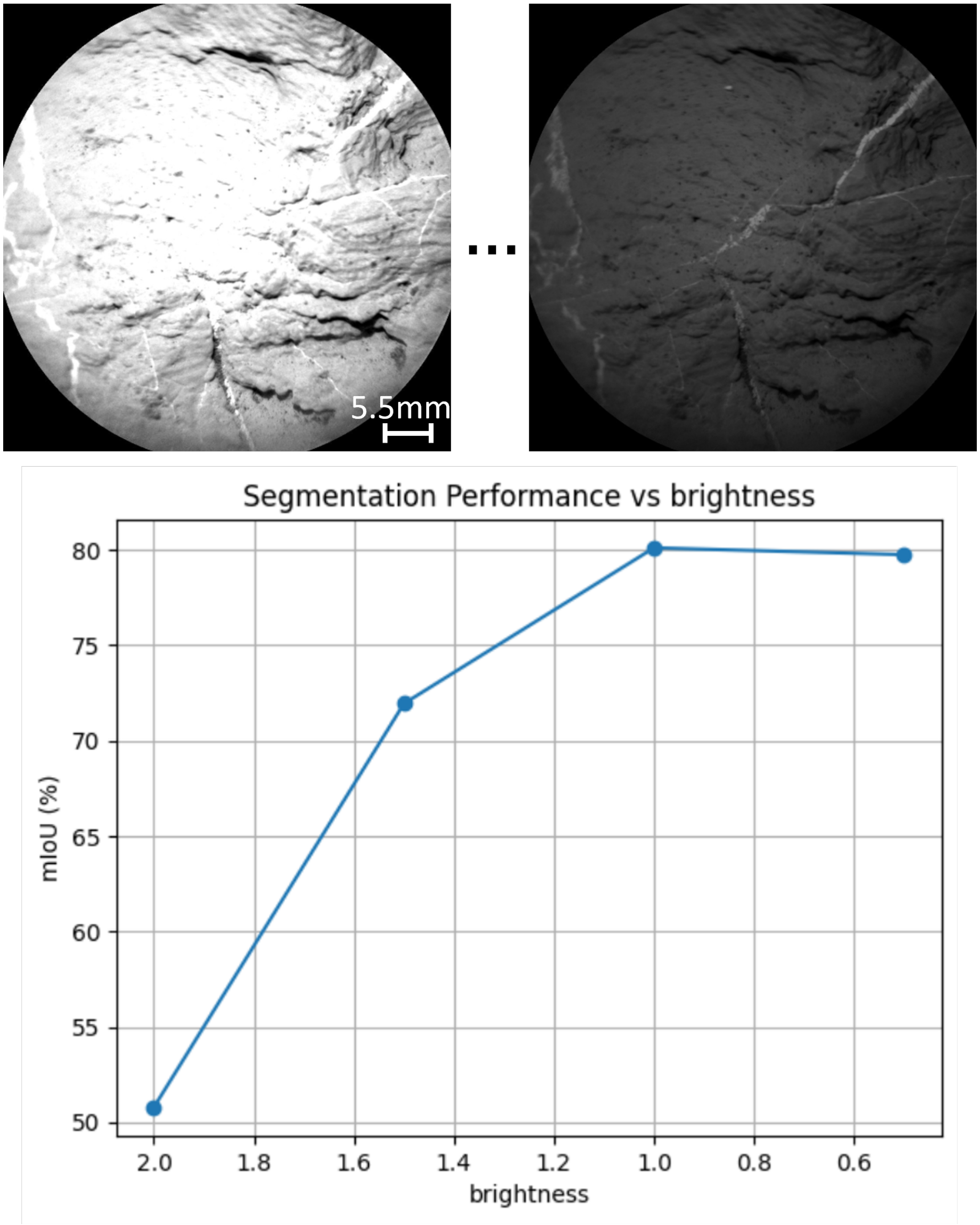


**Figure 2.** *Sensitivity of segmentation performance to changes in lighting conditions. Lighting conditions are synthetically modified using the OpenCV Cv2.ConvertScaleAbs method, where pixel intensities are adjusted according to* $newPixel = \alpha \times oldPixel + \beta$. *In our experiments,* $\alpha$ *is varied from 2.0 to 0.5, while* $\beta$ *is fixed at 0. The original RMI corresponds to* $\alpha = 1$, $\beta = 0$. *The top panels illustrate examples of this transformation. Top left: Example of an RMI with* $\alpha = 2.0$ *(increased brightness/contrast). Top right: example of RMI with* $\alpha = 0.5$ *(reduced brightness/contrast). The bottom panel shows the mean Intersection over Union (mIoU) of the best performing model as a function of a simulated lighting factor.*
